# Uptake, Translocation, and Stability of Oxytetracycline and Streptomycin in Citrus Plants

**DOI:** 10.3390/antibiotics8040196

**Published:** 2019-10-27

**Authors:** Fuad Al-Rimawi, Faraj Hijaz, Yasser Nehela, Ozgur Batuman, Nabil Killiny

**Affiliations:** 1 Department of Plant Pathology, Citrus Research and Education Center, IFAS, University of Florida, 700 Experiment Station Road, Lake Alfred, FL 33850, USA; 2 Chemistry Department, Faculty of Science and Technology, Al-Quds University, Jerusalem 90612, Palestine; 3 Department of Plant Pathology, Southwest Florida Research & Education Center, IFAS, University of Florida, 2685 State Road 29 North, Immokalee, FL 34142, USA

**Keywords:** Huanglongbing, oxytetracycline, streptomycin, antibiotic, translocation, citrus

## Abstract

Huanglongbing (HLB), or citrus greening, is the most destructive disease to the citrus industry. In Florida, it is caused by the bacterium, *Candidatus* Liberibacter asiaticus (*C*Las) and is transmitted by the Asian citrus psyllid, *Diaphorina citri*. Recent studies suggested that antibiotics could inhibit the growth of the *C*Las pathogen *in planta*. In the current study, we investigated the uptake and translocation of oxytetracycline and streptomycin in citrus seedlings. Oxytetracycline and streptomycin were delivered via root and stem and their level in various tissues was monitored using enzyme-linked immunosorbent assay (ELISA). Oxytetracycline and streptomycin were detected in the leaves, xylem, phloem, and root after root drench and stem delivery. High levels of antibiotics were detected in the roots after root drench, whereas high levels of antibiotics were detected in the canopy after stem delivery. The level of oxytetracycline detected in the phloem, xylem, and leaves after root drench was higher than that of streptomycin. Whereas the level of streptomycin in root was higher than that of oxytetracycline, indicating that streptomycin was bound to the xylem tissues. Oxytetracycline and streptomycin were detected in the phloem, xylem, leaves, and root tissues thirty-five days after the root incubation in 200 µg·mL^−1^ solution. These results demonstrated that oxytetracycline and streptomycin were relatively stable and could inhibit *C*Las growth for a couple of months in citrus trees. Observations reported in this study regarding the distribution and stability of oxytetracycline and streptomycin in citrus plants could be useful for designing an effective program for the control of HLB disease using antibiotics.

## 1. Introduction

Citrus is one of the major fruit crops in the world with an annual global production of about 100 million metric tons [1]. Citrus fruits are widely consumed for their health benefits; they are low in fats and rich in fiber, carotenoids, vitamins, minerals, and flavonoids. Unfortunately, citrus greening disease, which is also known as Huanglongbing (HLB), is currently threatening the citrus industry globally. *Diaphorina citri* Kuwayama, Asian citrus psyllid, is a global pest of citrus and is the vector of “*Candidatus* Liberibacter” pathogens responsible for causing HLB disease [2]. *D. citri* transmits the HLB pathogen while feeding on citrus phloem sap. HLB is currently considered the most destructive disease of citrus and has resulted in tree decline and loss of production in many regions [3]. *D. citri* was first discovered in Palm Beach County, Florida, on orange jasmine—*Murraya paniculata* (L.) Jack. (Rutaceae) in 1998—and it then spread to all citrus producing regions in the state [4]. The first incidence of HLB in the USA was reported in south Miami in August 2005. Currently, the HLB disease is endemic throughout the state and has significantly reduced citrus production. 

To date, there is no cure for HLB and most cultivars are susceptible to this destructive disease. Management of the HLB disease mainly depends on the control of the Asian citrus psyllid using a wide range of insecticides. The use of insecticides is essential in management of the disease since it reduces the number of insects in the field, protects non-infected and newly planted trees, and prevents repetitive inoculations by psyllids. However, the heavy use of insecticide negatively impacts human health, beneficial insects, and the environment [5]. In addition, the extensive use of insecticides can induce insecticide resistance in *D. citri* [6]. Enhanced nutritional programs (ENPs) were proposed to mitigate HLB symptoms in infected trees and maintain their productivity. The ENPs were initially used in China after the first discovery of the HLB [7]. ENPs usually consists of essential micronutrients such as manganese, copper, and zinc. Other ingredients such as salts of phosphite and salicylate were also added to the ENPs mixtures [7]. However, a two-year field study showed that the ENPs were not able to maintain tree health, fruit quality, and productivity [7]. In addition, there is a major concern about the use of ENP strategies because they could increase the spread of the *C*Las bacterium by increasing the lifespan of the infected trees [7]. Consequently, removal of infected trees was proposed to reduce *C*Las inoculum; however, this strategy has not been widely accepted by growers because it is expensive [4]. In addition, removal of infected trees is difficult to implement in heavily infected regions such as Florida, where more than 90% trees grown in the state are estimated to be infected.

A previous greenhouse study showed that thermotherapy was effective against the HLB pathogen. However, this technology is time consuming and expensive [8]. In addition, the use of this technology in the field did not show any promising results because thermotherapy did not significantly reduced the *C*Las titer in the roots as it did in the canopy [8]. Due to the difficulty managing HLB disease, development of tolerant citrus cultivars would be the best method for future HLB control. Unfortunately, development of tolerant cultivars is a big challenge because most of the current citrus cultivars are susceptible to HLB [9]. Consequently, the use of antibiotics could be an effective strategy for the control of HLB. 

The use of antibiotics for the control of HLB was initiated in the 1970s after the causal agent of HLB was suggested to be a microbial pathogen [8]. Early studies in the field showed that direct injection of tetracycline into the trunks of *C*Las-infected trees significantly reduced HLB symptoms [10,11,12,13]. In a greenhouse study, Capoor and Thirumal [14] showed that stem delivery of antibiotics was more efficient than foliar sprays and application under the bark . Application of achromycin (0.25–1.5 g/plant) by stem injection resulted in the recovery of all treated sweet orange seedlings [14]. Unfortunately, the use of oxytetracycline was discontinued because oxytetracycline is only a bacteriostatic and it has to be applied frequently, which could result in phytotoxicity [15].

Recently, the use of antibiotics for the control of HLB disease has regained interest by many growers and scientists due to significant losses in the citrus industry. For example, penicillin and streptomycin were found to be very effective in eliminating the *C*Las titer and rescuing of HLB-infected plants [16]. In addition, it was reported that ampicillin, carbenicillin, penicillin, cefalexin, rifampicin, and sulfadimethoxine were effective against *C*Las [15]. Greenhouse and field studies also showed that trunk injection of penicillin G significantly reduced the *C*Las titer in Ray Ruby grapefruit plants, without any adverse effect on the citrus native bacteria or root decay [17]. Trunk injection of penicillin, streptomycin and oxytetracycline hydrochloride resulted in excellent control of HLB [18]. In addition, antibiotics were found to be more effective in controlling HLB disease than plant defense activators like salicylic acid and oxalic acid [18]. Doud et al. [19] revealed via a graft-based assay that several antimicrobials such as aluminum hydroxide, nicotine, D, L-buthionine sulfoximine, and surfactin from *Bacillus subtilis* were effective against *C*Las. 

Antimicrobials such as streptomycin and oxytetracycline have been successfully used for the control of several plant disease since the 1950s [20]. Oxytetracycline has been registered for control of fire blight caused by *Erwinia amylovora* on peach and nectarine and for control of bacterial spot caused by *Xanthomonas arboricola* on peaches [21]. Oxytetracycline has also been used for the control of phytoplasmas, which cause the lethal yellow diseases in coconut palm and elm trees, as well as for the control of *Pseudomonas* spp. and *Xanthomonas* spp. on vegetables [21]. Unfortunately, the emergence of streptomycin resistant *E. amylovora*, limited the use of antibiotics in agriculture [21].

Oxytetracycline hydrochloride, oxytetracycline calcium, streptomycin sulfate have been approved for the treatment of HLB-infected trees in Florida since 2016 [22]. Consequently, investigating the uptake, distribution, and persistence of these antibiotics in citrus is necessary for better control of the *C*Las pathogen. In this study, we investigated the uptake and distribution of oxytetracycline and streptomycin in citrus plants via root and stem delivery. In addition, we tracked the residual level of the administered antibiotic in plant tissues for thirty-five days.

## 2. Results 

### 2.1. Percentage Recovery of Oxytetracycline and Streptomycin

The recovery of oxytetracycline and streptomycin from spiked tissues was 85.6 ± 8.7% and 89.7 ± 9.6%, respectively. No oxytetracycline or streptomycin was detected in controls or blank samples. The high percentage recovery indicated that our extraction method was efficient for the extraction of oxytetracycline and streptomycin.

### 2.2. Translocation of Streptomycin and Oxytetracycline in Citrus Seedlings via Root Drench and Stem Delivery

**Figure 1:**
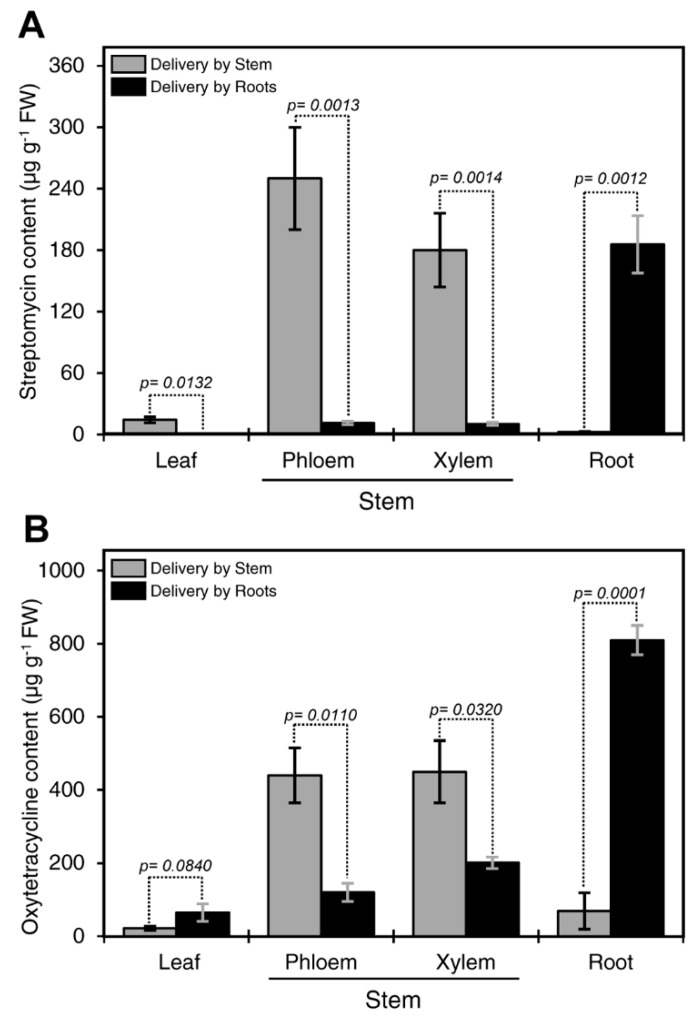
Comparison between root and stem delivery. Streptomycin content in various tissues in citrus plants treated with 200 µg·mL^−1^ streptomycin solution for 24 h via root or stem delivery methods (**A**). Oxytetracycline content in various tissues in citrus plants treated with 200 µg·mL^−1^ oxytetracycline solution for 72 h via root or stem delivery methods (**B**). For these analyses, the stem bark was dissected into the outer bark tissue (representing the phloem) and the inner bark (representing the xylem). Data are the means ± SD of five biological replicates. Columns with a *p-*value less than 0.05 indicate statistically significant differences between root and stem delivery method by Student’s *t*-test.

Figure 1A shows streptomycin residuals in different plant parts after incubation in antibiotic solution for 24 h. Streptomycin was detected in all plant tissues. The highest concentration was detected in the root, followed by phloem and xylem after root delivery (Figure 1A). Whereas, the highest level of streptomycin was detected in the phloem and xylem, followed by the leaves, and the root after stem delivery (Figure 1A). Low levels of streptomycin (<0.3 µg·g^−1^) were detected in leaves regardless of the delivery method.

We also assessed translocation dynamics of oxytetracycline by comparing between the stem and root delivery. The levels of oxytetracycline in the xylem and phloem after stem delivery were significantly higher than those obtained after root delivery (Figure 1B). Whereas, the levels of oxytetracycline in the root after stem delivery were significantly lower than those obtained via root delivery (Figure 1B). No significant differences were observed in the levels of oxytetracycline in the leaves via root or stem delivery (Figure 1B). The highest concentration of oxytetracycline was detected in the root followed by the xylem, phloem, and leaves after root delivery (Figure 1B). 

### 2.3. Stability of Streptomycin and Oxytetracycline in Citrus Plants after Root Drench

**Figure 2:**
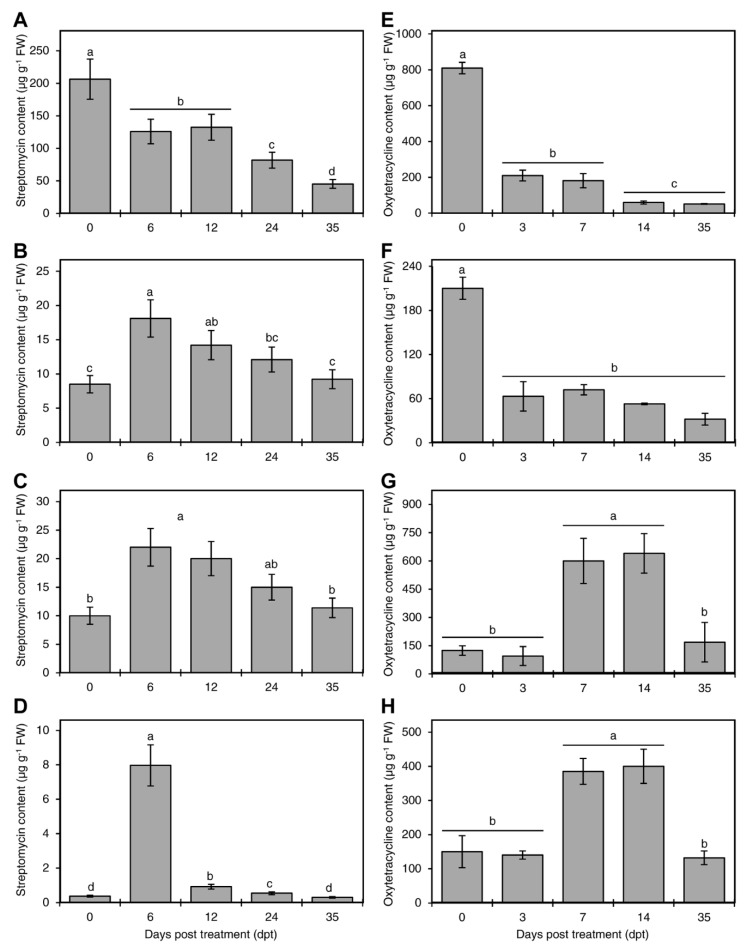
Persistence of streptomycin and oxytetracycline in citrus plants. The level of streptomycin at 0, 6, 12, 24 and 35 day-post-treatment in the root (**A**), xylem (**B**), phloem (**C**), and leaves (**D**) of Mexican lime as measured by ELISA after incubation in 200 µg·mL^−1^ streptomycin solution for 24 h via root drench method. The level of oxytetracycline at 0, 3, 7, 14, and 35 day-post-treatment in the root (**E**), xylem (**F**), phloem (**G**), and leaves (**H**) of Mexican lime as measured by ELISA after incubation in 200 µg·mL^−1^ oxytetracycline solution for 72 h via root drench method. Plant roots were washed with distilled water after treatments and the plants returned to their original soil until analysis. For these analyses, the stem bark was dissected into the outer bark tissue (representing the phloem) and the inner bark (representing the xylem). Data are the means ± SD of five biological replicates. Columns with different letters indicate statistically significant differences by Tukey HSD (*p* < 0.05).

The level of streptomycin declined with time in treated plants; however, it was still detectable in all plant tissue 35 (dpt) (Figure 2A–D). The level of streptomycin in the roots showed a continuous decline with time (Figure 2A). The level of streptomycin in the xylem and phloem followed a similar trend; it peaked at 6 dpt and declined thereafter (Figure 2B,C). The level of streptomycin in the leaves significantly increased from 0 to 6 dpt and declined thereafter (Figure 2D).

The level of oxytetracycline also declined with time in treated plants; however, it was still detectable in all plant tissue 35 (dpt) (Figure 2E–H). The level of oxytetracycline in the roots—and the xylem (Figure 2E,F)—showed a continuous decline with time. The level of oxytetracycline in the phloem peaked after 7 and 14 days and then declined thereafter (Figure 2G). The trend of oxytetracycline in leaves was similar to that observed in the phloem (Figure 2H).

### 2.4. Translocation of Oxytetracycline versus Streptomycin 

**Figure 3:**
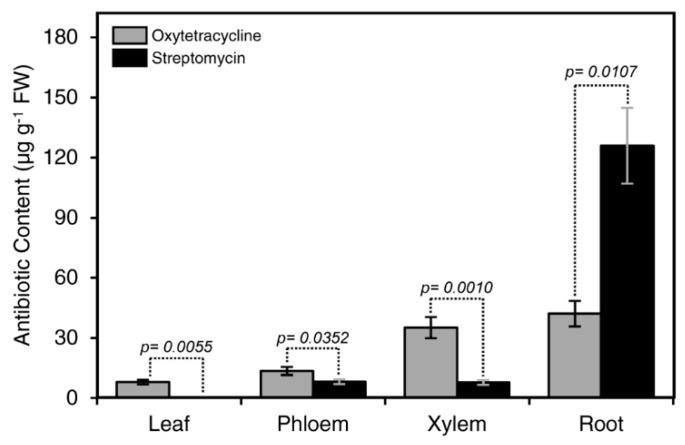
Comparison between streptomycin and oxytetracycline translocation in citrus plants. Plants were incubated in oxytetracycline or streptomycin solution (200 µg·mL^−1^) for 16 h and analyzed by ELISA. Columns with a *p-*value less than 0.05 indicate statistically significant differences between oxytetracycline and streptomycin by Student’s *t*-test.

The concentration of streptomycin in the root of treated seedlings was higher than oxytetracycline (Figure 3), indicating that streptomycin was highly bound to the surface of the root. On the other hand, the level of oxytetracycline in the xylem, phloem, and leaves was higher than that of streptomycin (Figure 3), indicating higher translocation of oxytetracycline.

## 3. Discussion

### 3.1. Translocation of Oxytetracycline and Streptomycin 

Oxytetracycline and streptomycin were detected in the xylem, phloem, and leaves of citrus seedlings after their roots were incubated in oxytetracycline or streptomycin solution. These results indicated that oxytetracycline and streptomycin were taken up by the xylem and translocated to other tissues. The highest concentration was found in the root, followed by the xylem, phloem, and leaves after root drench. The presence of oxytetracycline and streptomycin at high concentration in the phloem suggested that they could be effective against *C*Las, since it resides in the phloem. Although spiroplasmas and phytoplasmas were shown to be sensitive to a wide range of antibiotics, only oxytetracycline was effective against these agents in planta, indicating that it is highly translocated in the phloem [23]. Consistent with our results, oxytetracycline was also detected in alfalfa leaves after root incubation in oxytetracycline solution for few hours [24].

In agreement with our results, streptomycin was absorbed by the stems of bean seedlings and translocated to upper leaves in sufficient amounts to reduce the effect of the halo blight pathogen [25]. Streptomycin was also detected in the leaves of peach seedlings after their roots were kept in streptomycin solution [26]. Surprisingly, streptomycin was taken up by the cut shoots but not through the roots of broad beans, whereas it was translocated via both cut shoot and rooted tomato plants [27]. 

### 3.2. Stem versus Root Delivery

Our results showed that the levels of oxytetracycline in the phloem and xylem of citrus seedlings after stem delivery were higher than those obtained after root delivery. Whereas, the level of oxytetracycline in the root after root drench was higher than those measured in the root after stem delivery. Similar results were also observed with streptomycin. The result indicated that downward movement of oxytetracycline and streptomycin was limited. 

In agreement with our results, no antibiotic activity was detected in the small roots of sweet orange citrus trees that were injected with 10–30 g of oxytetracycline/tree via trunk injection, indicating limited downward movement [28]. Twigs and leaves showed high antibiotic activity, indicating higher upward movement of oxytetracycline most probably via xylem vessels [28]. Lee et al. (1982) also showed that oxytetracycline activity was high in canopy following trunk injection, whereas roots showed low or no activity. On the other hand, high antibiotic activity was observed in the roots after drench treatment [29]. The combination of drench and trunk injection resulted in high activity in the root and canopy of treated plants [29]. The antibiotic activity of penicillin G was detected in the root and canopy of the 2-year-old Ray Ruby grapefruit seedlings 24 h after trunk injection with 1.0 g/tree [17]. Interestingly, the activity of penicillin G against *Bacillus subtilis* was only observed in the canopy, but not in the roots, of the 7-year-old Ray Ruby grapefruit trees 24 h after injection with 6.0 g/tree [17]. The previous results indicated that downward movement of penicillin G could be limited in mature trees. In the same manner, air pressure injection of 1 L of water containing 6 g of oxytetracycline into the trunk of coconut trees (3–5 m height) resulted in high oxytetracycline concentration (20 µg·g^−1^ fresh weight) in the leaves two days after injection, whereas the concentration was less than 1 µg·g^−1^ fresh weight in the roots [30]. Soil drenches, foliar spray, and implantation of solid tablets of oxytetracycline produced no detectable residues in the leaves [30]. In the same manner, uptake of streptomycin through cut stems of peach seedlings was higher than root drench [26]. Recent studies showed that foliar application of oxytetracycline produced undetectable or mild levels of oxytetracycline residue in citrus leaves and did not reduce the *C*Las titer [31,32]. On the other hand, trunk injection of oxytetracycline (0.05 g/tree) significantly reduced the *C*Las titers to undetectable levels and produced high levels of oxytetracycline (0.68–0.73 µg/g fresh tissue) in 3-year-old Valencia sweet orange trees [32]. 

Although application of oxytetracycline and streptomycin through root drench resulted in high levels in the root, which could be effective against *C*Las, high levels of these antibiotics in the root could negatively affect root growth and beneficial bacteria in the rhizoplane. Daniels [23] showed that oxytetracycline activity was present in the root and the leaves of kidney beans after injection of 0.1 mL (1–10 mg mL^−1^) into the stem. The presence of oxytetracycline activity in the root suppressed the nodulation of treated plants, decreased the total number of bacteria in the rhizoplane, and increased the fungal populations and the number of oxytetracycline resistant bacteria in the rhizoplane [23]. A significant inhibition of root growth (up to 85%) was also reported in alfalfa plants after root incubation in 0.02 mM oxytetracycline solution [33]. Recently, Ascunce et al. [34] also showed that trunk injections of penicillin could affect the bacterial community structure in citrus trees. 

### 3.3. Stability of Oxytetracycline and Streptomycin in Citrus Seedlings

Oxytetracycline and streptomycin were still detectable in all tested tissues thirty-five days after treatment, indicating that these antibiotics are relatively stable in citrus plants. The level of oxytetracycline in the root and xylem declined with time, indicating a translocation of this antibiotic to other tissues. Whereas, the level of oxytetracycline in leaves and phloem continued to increase until the second week, and then declined thereafter. The decline of streptomycin was similar to that of oxytetracycline. These results suggested that the stem phloem and the leaves were the final destination of oxytetracycline and streptomycin in citrus after root delivery.

In agreement with our results, the activity of oxytetracycline was still detected (2−3 µg·g^−1^) in leaves thirty-six days after injection of 6 g into the trunk of coconut tree [30]. The activity of oxytetracycline also persisted in the twigs and leaves of sweet oranges 3−8 months after trunk injection of 10−30 g/tree [28]. The observed half-life of oxytetracycline in coconut palm trees was estimated to be 2−3 weeks [30,35], whereas it was 3−4 days in aster plant [36]. These results indicated that the stability of oxytetracycline varies among plant species. In addition, the long history of effective use of streptomycin and oxytetracycline for the control of several plant diseases, indicated that these antibiotics were relatively stable in plants [20,21]. If oxytetracycline and streptomycin are to be used in commercial citrus groves, then their residues in harvested fruits should be determined. Consequently, a proper wait time should be allowed between the application of antibiotics and harvest date (i.e., preharvest interval) in order to achieve low levels of antibiotics in harvested fruits. In fact, a recent study showed that trunk injection of oxytetracycline could result in low levels (202 μg/kg) of this antibiotic in citrus fruit [37]. The detected levels of oxytetracycline in citrus fruits were far below the minimum inhibitory concentration for gut flora, indicating negligible impacts on gut flora [37].

### 3.4. Translocation of Oxytetracycline versus Streptomycin

Our results showed that the level of streptomycin in the root after root drench was higher than oxytetracycline, indicating that streptomycin was highly bound to the surface of the root. Whereas, the level of oxytetracycline in the xylem, phloem, and leaves was higher than that of streptomycin, indicating oxytetracycline has a higher translocation rate than streptomycin. Accumulation of streptomycin was also reported in lower tissues of peach seedlings and tomato plants after incubation of their roots in streptomycin solution [26,27]. Accumulation of streptomycin (positively charged due to the presence of the two guanido groups) in lower tissues indicated that it was bound to the xylem surface, which is negatively charged [26]. The decrease of streptomycin levels from base to apex after root incubation and the decrease in its amounts in solutions after addition of peach-leaf tissues showed that streptomycin binds and accumulates in plant tissues [26]. This accumulation also indicated that saturation of the incubated tissues was needed before upward movement [27]. The uptake of streptomycin the cut shoots, but not through the roots of broad beans, indicated that it could be bound to the root tissues [27]. The previous observations indicated that streptomycin binding to the plant tissues could reduce its translocation rate.

Although antibiotics have been successfully used for the control of several plant disease since 1950s, their use have been challenged by several factors such as phytotoxicity at higher doses, inefficiency at low doses, and high cost [38]. In addition, evolution of streptomycin and tetracycline resistance in plant-pathogenic bacteria has become a big problem where these antibiotics have been used for several years [38]. Furthermore, improper application of antibiotics using air blast spraying system could affect the microbiome in plants and agriculture, and increase the resistance of human- and animal-pathogenic bacteria [38]. Consequently, we think that trunk injection is an efficient method for the delivery of these antibiotics and it could reduce the amount of applied antibiotics, minimize the drift of antibiotics in the environment, and decrease the chance for the development of human-pathogenic bacteria. In conclusion, the value of the use of streptomycin and oxytetracycline for the control of plant disease has decreased due to the development of antibiotic resistance in the plant-pathogenic bacteria; however, these antibiotics are still important for the control of critically important diseases. 

## 4. Materials and Methods

### 4.1. Plant Materials

Healthy Mexican lime (*Citrus aurantifolia*) seedlings were used in this study. Seeds were purchased from Lyn Citrus Seed, Inc. (Arvin, CA, USA) and individually potted in plastic cones (20 × 4 cm) containing Sungro professional growing mix (Sungro Horticulture, Agawam, MA, USA). Germinated seedlings were kept in a greenhouse (28 ± 1 °C, 60 ± 5% relative humidity, L16:D8 h photoperiod) at the Citrus Research and Education Center (CREC), University of Florida, Lake Alfred, Florida. Seedlings were watered twice weekly. All seedlings were about three months old and about 15 ± 5 cm tall.

### 4.2. Uptake of Antibiotics by Root Drench

**Figure 4:**
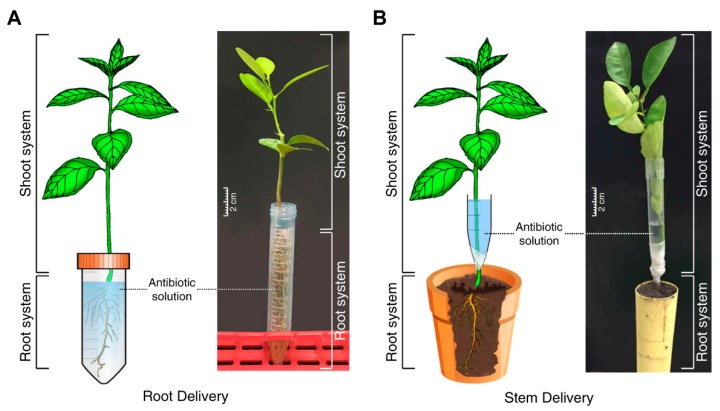
Delivery of streptomycin and oxytetracycline into 3-month-old citrus seedlings. Root drench (**A**) and stem delivery (**B**).

Oxytetracycline and streptomycin sulfate was purchased from Fisher Scientific (Pittsburgh, PA, USA). Oxytetracycline and streptomycin solution (200 µg·mL^−1^) were prepared in water. Streptomycin is readily soluble in water, whereas oxytetracycline is slightly soluble in water and was hence dissolved with the aid of sonication. To study the uptake of oxytetracycline and streptomycin by citrus seedlings, the roots were cleaned off soil by washing under tap water, then blot-dried on paper towels and immersed in 5 mL of 200 µg·mL^−1^ oxytetracycline or streptomycin solution in a 15 mL plastic centrifuge tube (Figure 4A). Control plants were incubated in distilled water. Five seedlings were used for each treatment. At the end of incubation time, the plants were washed for 1 min with distilled water to remove any adsorbed antibiotics from the root surface. The plants were dried using Kim wipes and the concentration of oxytetracycline and streptomycin was determined by ELISA.

### 4.3. Stem Delivery

To study the translocation of oxytetracycline and streptomycin through the stem application, several shallow cuts were made in the stems of each seedling by scalpel. The cuts were made at the bottom of the stem (about 3 cm above the root). A large pipette tip (10 mL) was cut lengthwise and was placed around the cut area of the stem. The pipet was sealed up by wrapping it with Parafilm (Figure 4B). The pipette tip was filled with 5 mL oxytetracycline or streptomycin solution (200 µg·mL^−1^) and the roots were placed back into the soil and the plants were incubated for the desired time. Control plants were incubated in distilled water. Five seedlings were used for each treatment. At the end of incubation time, reservoir was removed, and the seedlings were washed, as described before, dissected, and analyzed using ELISA. 

### 4.4. Stability of Oxytetracycline and Streptomycin in Plant Tissues

To study the persistence of oxytetracycline and streptomycin in citrus seedlings, twenty-five citrus seedlings were incubated (via root drench) in 200 µg·mL^−1^ oxytetracycline solution for 72 h. At the end of the incubation time, the roots were washed with distilled water to remove any adsorbed oxytetracycline, and the plants were returned to their original pots and left for different time intervals (0, 3, 7, 14, and 35 days). Control plants were incubated in distilled water. At the end of each time interval, the plants were removed from the soil, washed with distilled water and dissected into four parts (root, stem phloem tissue, stem xylem tissue, and leaves) and kept at −20 °C until analysis. Hereafter, phloem and xylem tissue extracted from stem bark tissue will be referred to as “phloem” and “xylem”. To determine the stability of streptomycin, another twenty-five citrus seedlings were incubated (via root drench) in 200 µg·mL^−1^ streptomycin solution for 24 h. At the end of the incubation time, plants were washed with distilled water and repotted in their original pots and were left for different time intervals (0, 6, 12, 24, and 35 days).

### 4.5. Extraction of Oxytetracycline and Streptomycin from Plant Tissues

Plant tissues were ground with liquid nitrogen using a mortar and pestle and 100 mg of the homogenous sample was transferred to a 2 mL centrifuge tube. One mL aliquot of 0.1 M HCl/0.01 M EDTA solution was added to each tube and the sample was vortexed for 1 min followed by sonication for 30 min. The extraction procedures were repeated twice, and the samples were centrifuged at 12,000 rpm for 10 min at 20 °C. The supernatant was transferred into a new tube and kept at −20 °C until analysis. To determine the efficiency of our extraction method, 100 mg of citrus tissues (control plant) was spiked with 100 µL of oxytetracycline solution of 1000 µg·mL^−1^ or 20 µL of 1000 µg·mL^−1^ streptomycin. Control samples were spiked with distilled water. The antibiotics were extracted as described above.

### 4.6. ELISA Assay

Oxytetracycline and streptomycin ACCEL ELISA kits were purchased from Plexense, Inc., (Davis, CA, USA) and were used according to manufacturer’s instructions. The quantitation ranges of the oxytetracycline and streptomycin kit were (1.56–50 ng·mL^−1^) and (0.150–12.5 ng·mL^−1^), respectively.

### 4.7. Statistical Analysis

Data were analyzed using JMP 9.0 software (SAS, Cary, NC). The *t*-test (*p* < 0.05) was used to compare between the stem and root delivery. The *t*-test (*p* < 0.05) was also used to compare between the levels of oxytetracycline and streptomycin after root delivery. Analysis of variance (ANOVA) followed by post hoc pairwise comparisons using Tukey-Kramer honestly significant different test (Tukey HSD) were used to compare levels of oxytetracycline among 0, 3, 7,14, and 35 dpt (*p* < 0.05). Tukey test was also used to compare levels of streptomycin among 0, 6, 12, 24, and 35 dpt (*p* < 0.05).

## 5. Conclusions

Our results showed that oxytetracycline and streptomycin were translocated in citrus plants after root drench and stem delivery. The presence of oxytetracycline and streptomycin in the phloem of treated plants suggested that these antibiotics could be effective against *C*Las. Oxytetracycline and streptomycin were detectable in all tissues tested 35 dpt, indicating that they are relatively stable in citrus plants and could be effective against *C*Las for a few months. Future studies should determine the efficacy of these antibiotics against the *C*Las pathogen in the field.
